# One-Pot FDCA Diester Synthesis from Mucic Acid and Their Solvent-Free Regioselective Polytransesterification for Production of Glycerol-Based Furanic Polyesters

**DOI:** 10.3390/molecules24061030

**Published:** 2019-03-15

**Authors:** Deyang Zhao, Frederic Delbecq, Christophe Len

**Affiliations:** 1 Sorbonne Universités, Université de Technologie de Compiègne, Centre de Recherches de Royallieu, CS 60319, F-60203 Compiègne CEDEX, France; deyang.zhao@chimieparistech.psl.eu; 2 PSL Research University, Chimie ParisTech, CNRS, Institute of Chemistry for Life and Health Sciences, 11 rue Pierre et Marie Curie, F-75231 Paris CEDEX 05, France; 3 Ecole Supérieure de Chimie Organique et Minérale, 1 allée du Réseau Jean-Marie Buckmaster, F-60200 Compiègne, France; f.delbecq@escom.fr

**Keywords:** mucic acid dehydration, FDCA diesters, regioselective transesterification, poly-transesterification

## Abstract

A one pot-two step procedure for the synthesis of diethyl furan-2,5-dicarboxylate (DEFDC) starting from mucic acid without isolation of the intermediate furan dicarboxylic acid (FDCA) was studied. Then, the production of three different kinds of furan-based polyesters— polyethylene-2,5-furan dicarboxylate (PEF), polyhydropropyl-2,5-furan dicarboxylate(PHPF) and polydiglycerol-2,5-furandicarboxylate (PDGF)—was realized through a Co(Ac)_2_·4H_2_O catalyzed polytransesterification performed at 160 °C between DEFDC and a defined diol furan-based prepolymer or pure diglycerol. In parallel to polymerization process, an unattended regioselective 1-OH acylation of glycerol by direct microwave-heated FDCA diester transesterification led to the formation of a symmetric prepolymer ready for further polymerization and clearly identified by 2D NMR sequences. Furthermore, the synthesis of a more soluble and hydrophilic diglycerol-based furanic polyester was also achieved. The resulting biobased polymers were characterized by NMR, FT-IR spectroscopy, DSC, TGA and XRD. The morphologies of the resulted polymers were observed by FE-SEM and the purity of the material by EDX.

## 1. Introduction

**Scheme 1:**
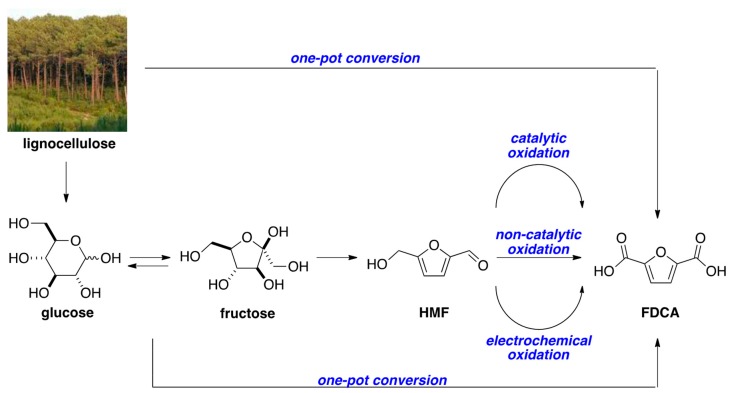
Different ways for the production of 2,5-furandicarboxylic acid starting from lignocellulose.

Furandicarboxylic acid (FDCA) is recognized as one of the twelve building-block chemicals by the US Department of Energy. This aromatic compound is easily synthesized through the dehydration of fructose to hydroxymethylfurfural (HMF) under acidic conditions and then peroxidation using various oxidants in one-pot one-step and one-pot two-step reactions (Scheme 1) [1,2,3,4]. The direct conversion of HMF to FDCA is technically feasible, but faces problems of cost, stability and availability.

Catalytic oxidation of HMF to FDCA using homogeneous catalysis was reported but these processes were less attractive as compared with heterogeneous catalysis due to the separation and recycling problems [5,6,7]. Modern heterogeneous catalysts such as nanosized bimetallic catalysts could also become a good alternative for the FDCA production [5,8,9,10]. Using homogeneous catalyst or heterogeneous catalyst the main limitation of the process remains the cost of HMF itself. In fact, this furanic intermediate is industrially produced in moderate yields by the dehydration of hexoses (fructose and glucose) isolated as sub-units from the depolymerization of polysaccharides such as cellulose or starch [11,12]. Nowadays, it appears important to find more efficient pathway to access FDCA analogs. One of the most promising routes should be the dehydration of mucic acid in the presence of strong mineral acid (H_2_SO_4_, H_3_PO_4_, HBr) [13] or *para*-toluenesulfonic acid (PTSA) [14]. Mucic acid offers the advantage of having two carboxylic acid groups.

FDCA is often employed as one of the components for the preparation of linear or branched polyesters showing relevant thermal and mechanical properties [15]. FDCA polyesters express good biocompatibility with the human body. These polymers are expected to be useful in a near future in the field of commodity thermoplastic engineering polymers like the well-exploited polyethylene terephthalate (PET). More exactly, FDCA is used to replace the *p*-terephthalic acid (PTA), which is overall an oxidation product of *p*-xylene, an aromatic compound issued from refined oil. Besides, PET is mainly composed of two Y-X-Y type monomers: PTA and ethylene glycol (EG). In case of EG, this diol is now a biobased building block generated by the biochemical transformation of D-xylose. The polymerization of FDCA or its counterparts with EG is already set up in industry for producing polyethylene-2,5-furan dicarboxylate (PEF) for commercial applications such as beverage bottles. From a physicochemical point of view PEF is a polyester of higher glass transition and structurally less linear than PET due to the angle of 129° formed between the two consecutive ester function hold by the furan rings. On the other hand, from a technical point of view, except the very classical melting polycondensation of FDCA mixed with a diol [16], to access to these polymers the furanic diacid need to be activated leading to the formation of its corresponding furan-2,5-dicarboxylic acid dichloride (FDCC) [17,18], but the process requires hazardous chlorination reagents such as phosphorous pentachloride or thionyl chloride. Once the diacyl chloride derivative is formed, it could react with various diols or diamines to form their respective polyesters and polyamides, but the main problem of FDCC accessibility is caused by the low solubility of FDCA in the common organic solvents. In comparison, the diesters of FDCA are easier to handle and far more soluble and could react with various diols in presence of catalysts such as tetraalkyl titanates [19,20,21], tin (II) 2-ethylhexanoate [22,23], antimony trioxide (III) [24,25,26,27,28,29,30] but always active at temperatures superior to 200 °C. A recent paper reported the use of 1,8-diazabicyclo [5.4.0]undec-7-ene (DBU) as an organocatalyst to perform the polytransesterification of FDCA dimethyl ester (DMFDC) in the presence of EG [31]. Another strategy to synthesize PEF involves the polymerization of a prepolymer named bis(hydroxyethyl)-2,5-furandicarboxylate (BHEFDC) [32]. This material is obtained by reaction of DMFDC immersed in a large excess of EG and heated at temperatures between 160 and 220 °C. The following polymerization must be carried out under reduced pressure to remove the released EG by-product. This kind of procedure is generally a source of possible degradation of the product and an energy consuming process. Generally, the solid polymers are obtained as dark brown solids only soluble in halogenated solvents such as trifluoacetic acid (TFA) or tetrachloroethane.

The first objective of this present work was to define greener reaction conditions to rapidly produce FDCA diethyl ester from mucic acid in ethanol, its transformation via metal-catalyzed transesterification into prepolymer counterparts using three really different polyols (EG, glycerol and α,α-diglycerol). Each prepolymer was allowed to react with one equivalent of the FDCA diester in the presence of cobalt (II) acetate to generate at a moderate temperature the target linear polymers. All compounds and materials reported in this present paper were studied by NMR and FT-IR spectroscopies. The polymeric materials were characterized by XRD, TGA and DSC analysis and the morphology of the solid were observed by FE-SEM.

## 2. Results and Discussion

**Scheme 2:**
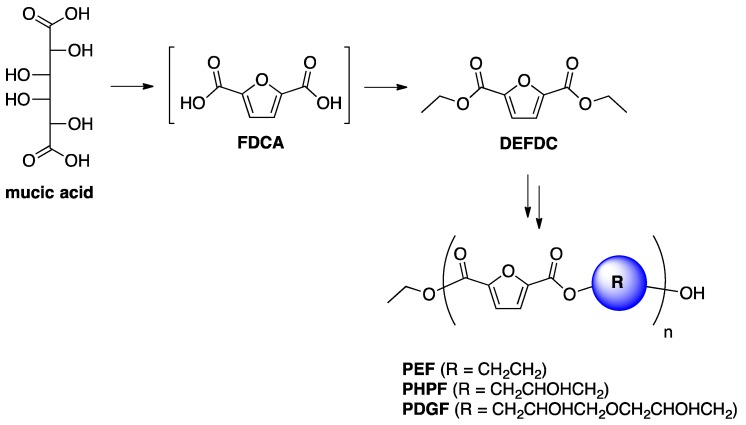
Entire synthetic routes to linear furanic polyesters from mucic acid.

In order to produce the furanic polyesters, a simple synthetic route was set up starting from mucic acid (Scheme 2). The main challenge remains the choice of mucic acid as the exclusive starting material for the production of furanic polyesters. In order to achieve the polymerizations, this method required the preparation of prepolymers through solvent-free transesterifications between the produced diethyl furan-2,5-dicarboxylate (DEFDC) and a chosen polyol in the presence of a transition metal diacetate.

### 2.1. Optimization of DEFDC Synthesis

For the purpose of carbon economy, the transformation of mucic acid into diethyl ester DEFDC was carried out in the presence of PTSA (4 equivalents) as acid catalyst in ethanol at 160 °C for 1 h. However, despite our efforts using a one-pot two-step procedure (formation of FDCA and then formation of the diester), only traces of the target diester DEFDC was obtained due to the low formation of FDCA. In this context, the decision of modifying our process was taken to optimize the FDCA production. Once produced in neat conditions by mixing mucic acid and acid catalyst, ethanol was added to the crude mixture containing the crude FDCA leading to the formation of the target compound (Scheme 2). Variations of: (i) the nature and loading of the acid catalyst; (ii) the temperature and (iii) the time of the reaction were studied. The effect of three catalysts (PTSA, methanesulfonic acid (MSA) and camphorsulfonic acid (CSA)) on the dehydration of mucic acid (1 mmol) was studied at different temperatures (140–170 °C) for 5–120 min keeping the amount of catalyst (1–4 eq) in a solvent-free reaction under conventional heating (Table 1). Initial studies were performed for mucic acid (1 mmol) and PTSA (1 eq) as a model reaction at 160 °C for 60 min. Under our conditions, the target FDCA was obtained in 32% yield. Variations of the catalyst loading showed that a maximal yield of FDCA (41%) was obtained in the presence of two equivalents of the acid catalyst (Table 1, entry 2). The use of more catalyst did not permit us to increase the productivity. At temperatures above 160 °C, the FDCA yield decreased (28% vs. 41%), probably due to some degradation and for temperatures below 160 °C, the production of FDCA was only 12% at 140 °C (Table 1, entries 16 and 17). Different times of reaction were tested with the optimized conditions and 60 min seemed to be the best one (Table 1, entry 2). Similar reaction profiles were obtained using MSA as acid catalyst (2 eq) at 160 °C with a shorter residence time (30 min vs. 60 min). Interestingly, CSA was not efficient for producing FDCA, despite its low pKa value of 1.2. In contrast with the results claimed by Taguchi et al. [33], our method did not afford the dialkyl 2,3-furandicarboxylate by-product.

Using the optimized conditions for the synthesis of FDCA (Table 1, entry 27), the second stage of the process was realized by adding dry ethanol (5 mL) to the crude material. Varying the reaction time (4–16 h) at 70 °C and 90 °C the maximal yield of DEFDC was obtained at 90 °C for 8 h (Table 2, entry 5).

### 2.2. Optimization of the Prepolymers Synthesis and Additional Regioselective Acylation of Glycerol

**Scheme 3:**
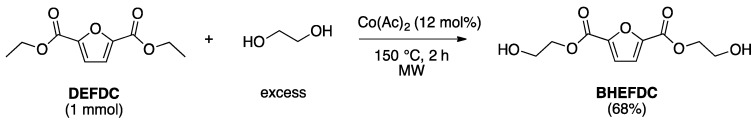
Synthesis of BHEFDC starting from DEFDC in the presence of Co(Ac)_2_ under microwave radiation.

After isolated and purified the diethyl ester DEFDC, the synthesis of three types of prepolymers such as BHEFDC, bis(2,3-dihydropropyl)-2,5-furandicarboxylate (BDHPFDC) and diglycerol analogue have been investigated. For BHEFDC, a study was realized under conventional heating. When heated at 150 °C for two hours DEFDC conversion was limited to 71% and the yield of BHEFDC never exceeded 60%. Keeping this temperature unchanged, by solely increasing the reaction time, a real enhancement of the BHEFDC yield has never been recorded. On the other hand, when the reaction was carried out at 170 and 190 °C, respectively, the DEFDC conversion went up progressively by extending both reaction time and temperature. Under the optimized conditions, at 190 °C, the almost complete conversion of DEFDC (95%) needs two hours to yield the prepolymer target in 67% yield. The effect of microwave irradiation on the above reaction was also investigated. After a short survey, batch microwave radiation gave similar results (68% yield) with conventional heating for 2 h but at a lower temperature (150 °C vs. 190 °C) (Scheme 3).

**Scheme 4:**
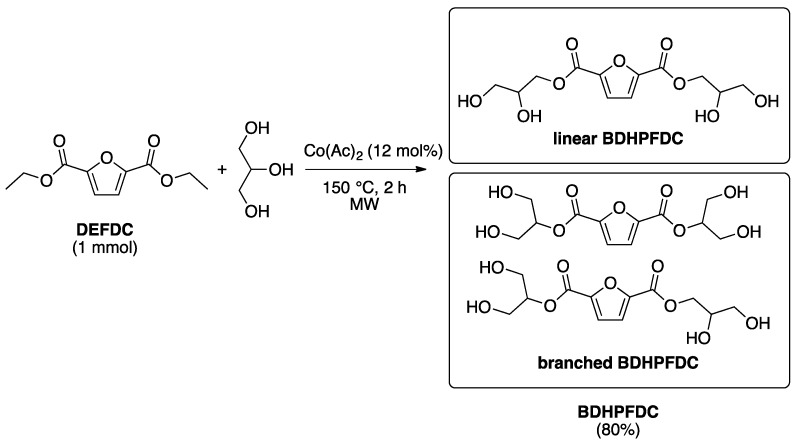
Synthesis of BDHPFDC starting from DEFDC in the presence of Co(Ac)_2_ under microwave radiation.

With a similar pKa for the two primary hydroxyl groups and the secondary group, the reactivity of glycerol did not permit regioselectivity without a sequence of protection/deprotection steps or a functionalization of the secondary hydroxyl group. Usually, there are only a few methods to direct the regioselective monoacylation of glycerol on the primary hydroxyl group. Recently, Slavko et al. developed a new catalyst-controlled polycondensation of glycerol using diacyl chlorides of terephtalic acids or adipic acids [34]. Herein, the authors employed an additional diarylboronic acid catalyst to form preferentially glycerol-derived linear polyester. Besides, it is well known that enzymatic controlled polymerization of glycerol is more or less able to generate linear and branched polyesters [35,36,37]. In order to produce the corresponding the tetrahydroxylated prepolymer BDHPFDC, our preceding procedure was applied at 160 °C for 13 h. Similarly, the batch microwave radiation permitted us to reduce the reaction time (2 h vs. 13 h) and the temperature (150 °C vs. 160 °C) with the synthesis of BDHPFDC in 80% yield (Scheme 4). It was notable that an unexpected efficient regioselective monoacylation of glycerol on its primary hydroxyl group was observed permitting the formation of linear polymer. To clearly determine the regioselectivity of our trans-esterification, 2D-NMR experiments were employed to estimate the selectivity of the reaction between the primary hydroxyl group and the secondary hydroxyl group furnishing the linear prepolymer and the branched prepolymers, respectively (Figure S1). After investigation, the amount of the branched dicarboxylates present in the solid was estimated at a maximum of 3%. In our opinion this quite regioselective BDHPFDC synthesis is probably promoted by the direct complexation of Co (II) on glycerol and the metal remained attached to all reaction intermediates until the transesterification processes were completed.

Applying the process to diglycerol as polyol was not successful and it was impossible for us to isolate the desired diol derivatives whatever the temperatures used (160 and 180 °C).

### 2.3. Optimization of Solvent-Free Poly Trans-Esterification and Solids Characterizations

**Scheme 5:**
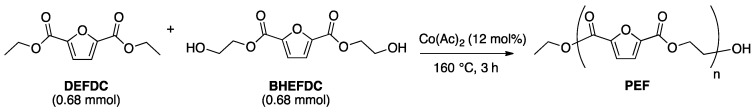
Synthesis of PEF in the presence of Co(Ac)_2_.

**Figure 1:**
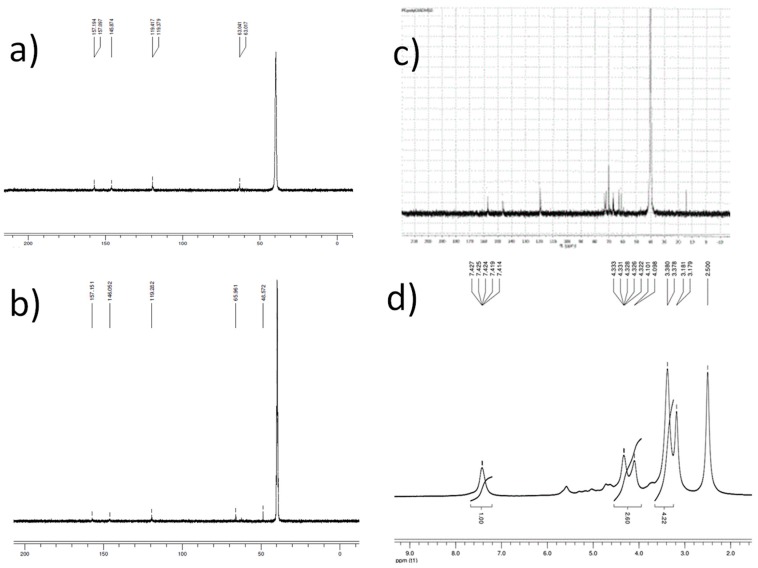
(**a**) ^13^C-NMR spectrum of PEF in DMSO-*d*_6_; (**b**,**d**) ^13^C- and ^1^H-NMR spectra of PHPF in DMSO-*d*_6_ and (**c**) ^13^C-NMR of PDGF measured in DMSO-*d*_6_.

Currently, our strategy involved to mix DEFDC (1 eq) with BHEFDC (1 eq), BDHPFDC (1 eq) and diglycerol (1 eq), separately in the presence of catalytic amount of Co(Ac)_2_ in solvent free conditions at 160 °C for 3 h. In several cases, dark brown solids or powders were isolated prior to further analysis. All materials were insoluble in the common organic solvents currently employed in steric exclusion chromatography (SEC) analysis. Starting from DEFDC and BHEFDC, the target polymer PEF was obtained (Scheme 5). The ^1^H-NMR spectrum of PEF corroborated the result reported in the literature [21,26] and showed in different samples studied at successive residence time the progressive disappearance of signal of methyl group at 1.36 ppm which is representative of the DEFDC ester moieties (Figure S2). The small peak located at 3.34 ppm was attributed to the primary alcohol of BHEFDC. The ^13^C-NMR spectrum of PEF was identical with that published [21,26] (Figure 1a). Apparently, one of the major defaults of this synthetic method is the absence of control regarding chain termination with an apparent suppression of the ethyl ester functions.

**Scheme 6:**
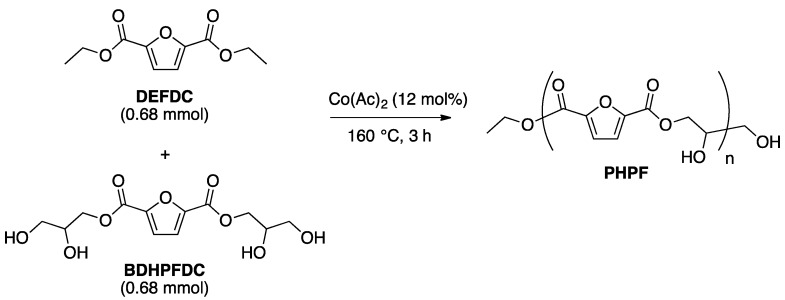
Synthesis of PHPF in the presence of Co(Ac)_2_.

Starting from DEFDC and BDHPFDC, the target polymer PHPF having a glycerol moiety was obtained (Scheme 6). The principal risk of this reaction was the possibility of forming branched polyesters due to the presence of two secondary hydroxyl groups on the glycerol-based prepolymer. However, compared to an already described non-linear polymer [28], our result is coherent with a linear structure. The signal of the primary alcohol of the prepolymer does not remained observable on the ^1^H-NMR spectrum of the sample. Moreover, the ^1^H-NMRs of the glycerol-based copolymer clearly showed bands between 3.17 and 3.38 ppm that match the -CH- of the secondary hydroxyl group of glycerol (Figure 1d). Another broad signal was also observable from 4.01 and 4.33 ppm that corresponds to the methylene groups of the polyol included in the polyester chains. The ^13^C-NMR spectrum of PHPF was also more simplified compared to its branched counterparts [28] showing only five peaks: 48.5, 65.9, 119.3, 146.0 and 157.1 ppm (Figure 1b).

Starting from DEFDC and diglycerol, a more usual poly transesterification was carried out under conventional heating at 160 °C for 3 h. Compared to the ^13^C-NMR spectrum of PHPF, the number of peaks was higher considering those initially expected, evidence of possible branching of the polymer chains (Figure 1c). The ^1^H-NMRs of this product material are also given (Figure S3). According to the results displayed in the COSY spectrum of BDHPFDC, some similarities can be found with spectra obtained from a sample of PDGF in DMSO-*d*_6_. Located in the center part of the corresponding spectrum grid four signals around 4.5 ppm in both directions form a square and are representative of the furan rings bound to diglycerol through a primary alcohol ester made of CH_2_ groups (Figure S4). Besides, at 3.6 and 5.1 ppm, there are two signals rather characteristic of furan moieties bonded with a secondary alcohol group that corresponds to the –CH- group of the diglycerol. Those results are evidence of the non-linear nature of the formed PDGF polymer.

In order to complete the spectrochemical analysis, FT-IR spectra of the three polymers PEF, PHPF and PDGF provided us important information about our materials (Figure S5). Thus, all characteristic vibration bands of PEF were detected in the spectrum and reported as follows: 2881 (λ CH_2_), 1717 (C=0), 1582 (λ C=C), 1271 (C-O), but also 963, 832 and 764 cm^−1^ which are the bending motions always associated with the 2,5-disubstituted furan ring. For the poly(2,5-furan dicarboxylic acid-co-glycerol) PHPF and PDGF, the material was analyzed and compared to FT-IR spectrum obtained from the branched polymer recently synthesized by Amarasekara et al. [28]. In the PHPF spectrum, it was possible to observe the characteristic bands of furan-based polyesters located respectively at 1733 (C=O), 1157 and 1276 cm^−1^ (C-O-C). Unfortunately, it was impossible for us to detect the presence of the typical -OH vibration bands. On the other side, all representative bands of the furan-based polyesters were effectively present. For PDGF, the bands of –OH vibrations were clearly visible around 3297 cm^−1^ and all other characteristic bands located at 2955, 1720, 1584 cm^−1^, but also 1128 and 1278 cm^−1^ attributed to the diglycerol moiety.

**Figure 2:**
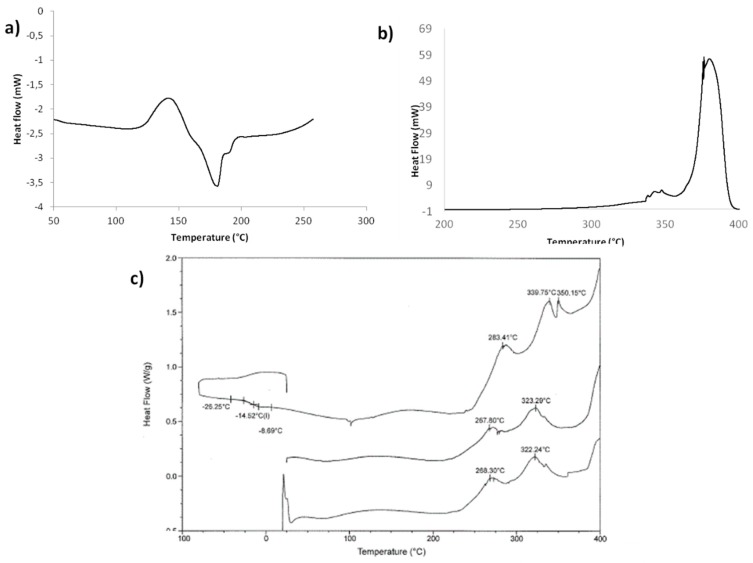
DSC thermograms of (**a**) PEF and (**b**) PHPF (**c**) PDGF after quenching their melted solution with liquid N_2_. Heating and cooling rate was 10 °C/min.

The DSC thermograms of our synthesized PEF, PHPF, and PDGF were measured (Figure 2). Due to a possible high crystallinity level, the melting point *T*m recorded for PEF gave a value a little bit inferior than the normal value of 211 °C generally encountered in the literature [10,15,19], but still superior to 183 °C. This result could be connected to the length of the polyester chain formed during the process. A normal crystallization exotherm was present in the thermogram and the material expressed a cold crystallization temperature Tc at 142 °C. In comparison, PHPF did not show an endothermic peak but rather two strong peaks located at 345 and 379 °C, respectively, in the thermogram. This result could be attributed as the first evidence of a possible modification of the amorphous structure of the material giving later a glassy appearance after a treatment at higher temperatures. For PDGF, the thermogram displayed at temperatures superior to 250 °C, two successive crystallization values of Tc observed at 267 and 322 °C. Curiously, after further warming cycles, a new peak started to appear at 350 °C. It is possible to attribute this phenomenon to degradation or rearrangements of the polymer backbone.

**Figure 3:**
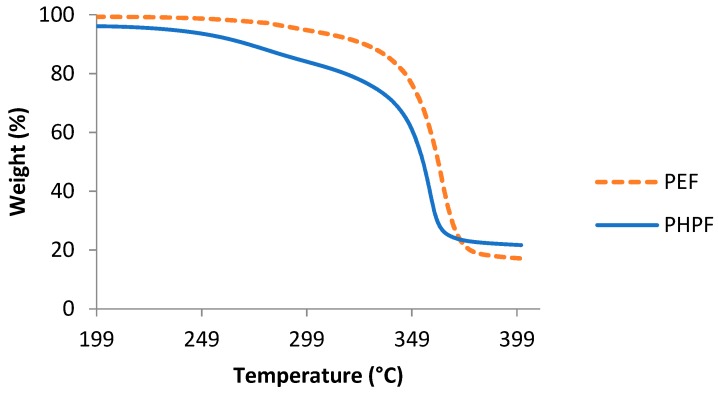
TGA traces curves of PEF and PHPF.

TGA measurements were recorded for two of our polymeric samples for evaluating the stability of our polymers at the higher temperature in air or under a nitrogen atmosphere (Figure 3). For PEF, the material is quite stable for temperatures above 220 °C, but around 380 °C the material lost about 60% of its total mass. This result is in accordance with literature [10,15,19]. For PHPF, the mechanism appeared to be more complicated. In fact, the glassy black solid already lost around 4% of its mass at 200 °C, but the DTGA curve clearly showed two peaks located at 290 and 380 °C, respectively (Figure S6). The first one could be representative of a partial dehydration of the polyester chain localized on the glycerol moiety followed by structural transformations that could occur at higher temperatures. Finally, both polymers undergo mass variations at temperatures below 400 °C, this result is also in agreement with the above DSC measurements.

**Figure 4:**
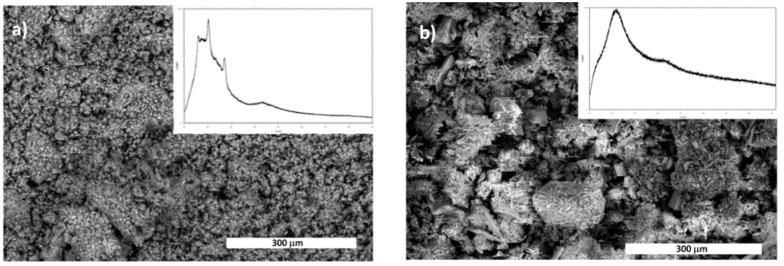
XRD diffractograms and SEM images of synthesized (**a**) PEF and (**b**) PHPF.

The crystal structures of PEF and PHPF were estimated by XRD (Figure 4). For PEF, the diffractogram displayed three representative sharp signals observed at 18, 22 and 28°, characteristic of a semi-crystalline material. On the contrary, the glycerol-based PHPF was completely amorphous and did not show a real crystallinity pattern. Unsurprisingly, PDGF was also an amorphous material and did not display peaks or signals in the XRD analysis. FE-SEM observations of PEF powder morphology displayed some kind of fibrous and apparently porous network. In association with this result the EDX spectrum showed that the purified material was rich in carbon and oxygen, but some traces of cobalt salts remained entrapped inside the material. Conversely, in the PHPF photograph, it is possible to observe larger and entangled structures, unfortunately loaded with more cobalt traces, proven by the corresponding EDX spectrum (Figure S7). This result could be suggested by the presence of a great number of hydroxyl groups present on the surface of the material able to complex a certain amount of the catalyst.

In order to determine the sustainability of our processes, EcoScale has been calculated as a rapid metric [38] for the optimized syntheses of FDCA, DEFDC, BHEFDC and BDHPFDC. For the production of the polymers PEF, PHPF and PDGF with undetermined yields and molecular weights the EcoScale was not calculated. The values obtained using the Ecoscale tool allow us to rank our optimized experiments as follow: acceptable reactions conditions for the synthesis of FDCA with a total score of 68; inadequate reaction conditions for the synthesis of DEFDC with a total score of 32; excellent reaction condition for the synthesis of BHEFDC and BDHPFDC, with total scores of 75 and 81, respectively.

## 3. Experimental Section

### 3.1. General Information

Ethanol absolute (99.5%, extra dry), mucic acid (98%), diglycerol, ethylene glycol (99%, extra pure), cobalt (II) acetate tetrahydrate (Co(Ac)_2_, 4H_2_O; 98%) and methanesulfonic acid (MSA, 99% extra pure) were purchased from Acros Organics (Pittsburgh, PA, USA). Other reagents such as *p*-toluenesulfonic acid monohydrate (PTSA·H_2_O; 98%), furan-2,5-dicarboxylic acid (98%) and glycerol (reagent plus > 99% GC) were obtained from Alfa Aesar (Ward Hill, MA, USA) and Sigma Aldrich (Saint Louis, MO, USA). All chemicals were used as received without further purification.

Transesterifications were realized with a microwave apparatus (Monowave 300, Anton Paar, Graz, Austria) at 600 rpm for the desired time. The reaction vessel was purged three times with nitrogen. The temperature in the reaction vessel was monitored by means of an IR sensor and the vial was pressurized in regard to the normal vapor pressure of the mixture at defined reaction temperature.

Nuclear magnetic resonance (^1^H- and ^13^C-NMR) spectra were recorded on an Avance III 400 spectrometer (Bruker, Billerica, MA, USA) equipped with a BBFO probe operating at room temperature. Other sequences such as COSY (^1^H-^1^H), direct HSQC (^1^H-^13^C) and HMBC long range coupling (^1^H-^13^C) were applied on monomers BHEFDC and PDGF polymers. Chemical shifts (δ) are quoted in parts per million (ppm). Coupling constants are quoted in Hertz. Sometimes, comparison with already described compounds has been used to confirm the NMR peak assignments.

For high performance liquid chromatography (HPLC), each sample was analyzed separately by means of a Prominence HPLC system (Shimadzu, Kyoto, Japan). Unreacted mucic acid was detected with a low temperature evaporative light scattering detector (ELSD-LT II) and the products concentration was estimated with a UV-Vis detector (SPD-M20A) at a wavelength of 265 nm. The column used was a Grace (Columbia, MD, USA) Prevail C18 column (250 × 4.6 mm 5 μm). The mobile phase was MeOH-water (9:1) solution flowing at rate of 0.5 mL/min. The column oven temperature was set at 40 °C.

Differential Scanning Calorimetry (DSC) was conducted on a DSC 8MC apparatus (Mettler-Toledo, Columbus, OH, USA) using aluminium pans. Scans were conducted under nitrogen with a heating rate of 10 °C/min in the temperature range of 40–250 °C.

Thermogravimetric analysis (TGA) was carried out on an Setsys Evolution analyzer (ATG Setaram, Caluire, France) equipped with an aluminium cell, using aluminium pans to encapsulate the samples. Typically, samples were heated at a constant rate of 10 °C/min from room temperature up to 550 °C, under a helium flow of 50 mL/min. The thermal decomposition temperature was taken at the onset of significant (≥5%) weight loss from the heated sample.

FT-IR (ATR) spectra were recorded on FT-IR 4000 (Jasco, Tsukuba, Japan) in a range of 650 to 4000 cm^−1^.

FE- Scanning Electron Microscopy (SEM)- Energy Dispersive X-ray Diffraction (EDX) analysis of polymer powders was performed on a Quanta FEG 250 instrument (FEI, Hillsboro, OR, USA) equipped with a microanalysis detector for EDX from Bruker. SEM micrographs acquired in secondary electron mode were obtained at low vacuum, 15 Kv accelerating voltage and 10 mm working distance.

XRD experiments were recorded on a X’pert MRD diffractometer (Pan Analytic/Philips, Eindhoven, the Netherlands 40 Kv, 30 mA) using Cu Kα (λ = 0.151418 nm) radiation. Scans were performed over a 2θ range from 10 to 80, at step size of 0.018° with a counting time per steps of 5 s.

### 3.2. Synthesis and Characterization

#### 3.2.1. Synthesis of Diethyl Furan-2,5-Dicarboxylate (DEFDC)

A suspension of mucic acid (210.0 mg, 1.0 mmol) in the presence of MSA (192.0 mg, 2.0 mmol) was stirred at 160 °C for 30 min using an oil bath until the reaction mixture turned brown. Then, after cooling the mixture to 70 °C, dry ethanol (5 mL) was added and the mixture was heated progressively from 70 to 90 °C for additional 8 h. The reaction mixture was allowed to return to the room temperature, poured in water and extracted with ethyl acetate. The organic layer was separated, washed with aqueous NaHCO_3_ saturated solution. The organic liquid was isolated, dried on MgSO_4_ and evaporated in vacuum to afford a brown solid which was purified on silica gel column chromatography using a mixture of EtOAc-cyclohexane (1:1) as eluent to afford the target DEFDC as a white crystal (m = 61.5 mg; yield = 29% for two steps); Mp = 86 °C; ^1^H-NMR (CDCl_3_): δ 1.32 (t, 6H, *J* = 7.2 Hz, CH_3_), 4.33 (m, 4H, CH_2_), 7.13 (s, 2H, CH Furan ring) ppm; ^13^C-NMR (CDCl_3_): δ 14.3, 61.6, 118.2, 149.0, 158.0 ppm.

#### 3.2.2. Synthesis of Bis(Hydroxyethyl)-2,5-Furandicarboxylate (BHEFDC)

DEFDC (212.1 mg, 1.0 mmol) was mixed with EG (4.0 mL) in presence of 20.0 mg (0.08 mmol) of Co(Ac)_2_, 4H_2_O. The mixture was introduced in a 25 mL round bottom flask equipped with water separator and nitrogen inlet. The reaction was carried out at a selected temperature from 150 for 2 h under microwave radiation. Finally, the purification was realized as reported previously affording the compound as colored crystals [21] (m = 166.0 mg; yield = 68%); The material was hygroscopic; ^1^H-NMR (CDCl_3_): δ 3.87 (m, 4H, CH_2_), 4.37 (m, 4H, CH_2_), 4.59 (s, 2H, OH), 7.17 (s, 2H, CH Furan ring); ^13^C-NMR (CDCl_3_): δ 60.6, 67.2, 118.9, 146.4, 158.1.

#### 3.2.3. Synthesis of Bis(2,3-Dihydropropyl)-2,5-Furandicarboxylate (BDHPFDC)

DEFDC (212.1 mg, 1.0 mmol) was mixed with glycerol (3.0 mL) in presence of Co(Ac)_2_·4H_2_O (20.0 mg, 0.08 mmol). The mixture was introduced in a 25 mL round bottom flask equipped with water separator and nitrogen inlet. The reaction was carried out at a selected temperature from 150 for 2 h under microwave radiation. After performed the reaction, the sample tube was left at room temperature for 24 h, then a product started to recrystallize slowly from the viscous solution. The product was recovered by centrifugation after addition of 1 mL of cold water to the mixture. Then the collected crystal was dried in vacuum to afford the prepolymer as a white solid. (m = 243.3 mg; yield = 80%). Mp = 83 °C; FT-IR: 2850-2900; 1728 (C=O); 1279; 1230; 1154; 760 cm^−1^; ^1^H-NMR (DMSO-*d*_6_): δ 3.59 (m, 4H, CH_2_), 3.98 (m, 2H, CH), 4.24–4.40 (m, 4H, CH_2_), 7.32 (s, 2H, CH Furan ring); ^13^C-NMR (DMSO-*d*_6_): δ 62.0, 66.4, 69.3, 119.6, 146.2, 159.3; +ESI-TOF-MS m/z = 304 + 1 (M^+^H^+^).

#### 3.2.4. General Melt Polytransesterification

DEFDC (144.2 mg, 0.68 mmol) and 1 equivalent of BHEFDC (166.0 mg, 0.68 mmol), BDHPFDC (206.8 mg, 0.68 mmol) or diglycerol (112.9 mg, 0.68 mmol) were mixed with Co(Ac)_2_·4H_2_O (20.0 mg (0.08 mmol). The solvent free mixture was stirred at 160 °C under nitrogen atmosphere until all material melted for a minimum of three hours to complete the polymerization. Later the resulted dark brown mixture is allowed to return to the room temperature, each solid was triturated in MeOH, filtrated and dried in vacuum prior to physicochemical characterization.

For PEF: ^1^H-NMR (DMSO-*d*_6_): δ 7.4 (s, CH furan rings), 4.65 (s, CH_2_); ^13^C-NMR (DMSO-*d*_6_): δ 63.0 (CH_2_), 119.4 (CH), 145 (Cq Furan), 157.1 (Cq, C=O).

For PHPF: ^1^H-NMR (DMSO-*d*_6_): δ 7.42 (s, CH furan rings), 4.32 (s, CH_2_), 3.38 (CH); ^13^C-NMR (DMSO-*d*_6_): δ 48.5 (CH), 66.0 (CH_2_), 119.3 (CH), 146.0 (Cq Furan), 157.1 (Cq, C=O).

For PDGF: ^1^H-NMR (DMSO-*d*_6_): δ 7.42 (s, CH furan rings), 5.50–3.20 (m, CH_2_ and CH), 1.32 (m, CH_3_).

## 4. Conclusions

A new process for the synthesis of the diethyl ester of FDCA starting from mucic acid was developed in a one-pot two step procedure furnishing the target diester in 29% yield. Using this platform molecule, two prepolymers were produced by transesterification with ethylene glycol and glycerol and Co(Ac)_2_·4 H_2_O as catalyst in 68% and 80% yields, respectively. Then, different kinds of furan-based polyesters were produced using a solvent-free Co(Ac)_2_·4H_2_O catalyzed polytransesterification between FDCA diethyl ester and two polyol- derived prepolymers (PEF and PHPF). The glycerol-based linear polyester seems to be subject to some kind of further rearrangement causing reticulation of the material, when heated at temperatures above 370 °C. However, due to the increasing number of hydroxyl groups on the periphery of the material, the viscous resin of PDGF was clearly a water soluble material. This methodology should be extended to other biosourced diols or PTA-based prepolymers to access new copolymers of interest. The resulting linear PHPF and branched PDGF polymers could be also functionalized on their free hydroxyl group for future biological or medicinal applications.

## Figures and Tables

**Table 1:**
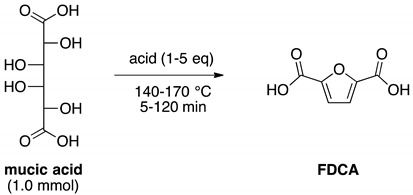
Dehydration of mucid acid in the presence of acid catalyst for the production of FDCA.

Entry	Acid	[Acid] [eq]	Temperature [°C]	Time [min]	Yield of FDCA [%] ^a^
1	PTSA	1	160	60	32
2	PTSA	2	160	60	41
3	PTSA	3	160	60	36
4	PTSA	4	160	60	34
5	PTSA	5	160	60	37
6	MSA	1	160	60	34
7	MSA	2	160	60	39
8	MSA	3	160	60	35
9	MSA	4	160	60	35
10	MSA	5	160	60	30
11	CSA	1	160	60	4
12	CSA	2	160	60	11
13	CSA	3	160	60	15
14	CSA	4	160	60	12
15	CSA	5	160	60	9
16	PTSA	2	140	60	12
17	PTSA	2	170	60	28
18	PTSA	2	160	5	8
19	PTSA	2	160	15	20
20	PTSA	2	160	30	30
21	PTSA	2	160	90	40
22	PTSA	2	160	120	37
23	MSA	2	140	60	15
24	MSA	2	170	60	25
25	MSA	2	160	5	8
26	MSA	2	160	15	30
27	MSA	2	160	30	39
28	MSA	2	160	90	35
29	MSA	2	160	120	32

^a^ The FDCA yield was calculated from liquid chromatography analysis with a calibration curve.

**Table 2:**
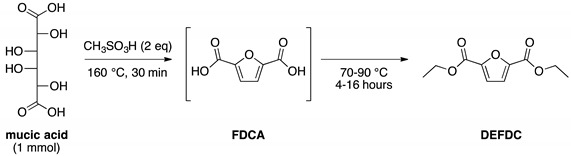
Production of DEFDC starting from mucic acid in a one-pot two steps reaction.

Entry	Temperature [°C]	Time [h]	Yield of DEFDC [%] ^a^
1	70	4	15
2	70	8	23
3	70	16	29
4	90	4	27
5	90	8	29
6	90	16	30

^a^ The DEFDC yield was calculated from liquid chromatography analysis with a calibration curve.

## Supplementary Materials

The following are available online. Figure S1: 2D-NMR sequences of BDHPFDC: COSY (^1^H-^1^H) and HMBC (^1^H-^13^C), Figure S2: ^1^H NMR of PEF in DMSO-*d*_6_ after complete polymerization, Figure S3: ^1^H NMR of PDGF after polymerization, Figure S4: Cosy (1H-1H) spectrum of PDGF, Figure S5: FT-IR spectra of (a) PEF; (b) PHPF and (c) PDGF, Figure S6: TG-DTG curves of (a) PEF and (b) PHPF, Figure S7: EDX spectra of (a) PEF and (b) PHPF.
