# Supplementary material for: One-Pot FDCA Diester Synthesis from Mucic Acid and Their Solvent-Free Regioselective Polytransesterification for Production of Glycerol-Based Furanic Polyesters
Source: Molecules. 2019 Mar 15;24(6):1030. doi: 10.3390/molecules24061030 (PMC6471091; doi:10.3390/molecules24061030)
Supplement: Supplementary file 1 [file molecules-24-01030-s001.pdf]

Electronic Supporting information

One-pot FDCA diesters synthesis from mucic acid and their solvent-free regioselective polytransesterification for production of glycerol-based furanic polyesters

Deyang Zhao,[a,b] Frederic Delbecq,[c] Christophe Len*[a,b]

[a] Sorbonne Universités, Université de Technologie de Compiègne, Centre de Recherches de Royallieu, CS 60319, F-60203 Compiègne cedex, France.

[b] PSL Research University, Chimie ParisTech, CNRS, Institute of Chemistry for Life and Health Sciences, 11 rue Pierre et Marie Curie, F-75231 Paris Cedex 05, France.

[c] Ecole Supérieure de Chimie Organique et Minérale, 1 allée du Réseau Jean-Marie Buckmaster, F-60200 Compiègne, France.

Fax: +33 (0)344 971 591; Tel: +33 (0)638 500 976; E-mail:christophe.len@chimieparistech.psl.eu

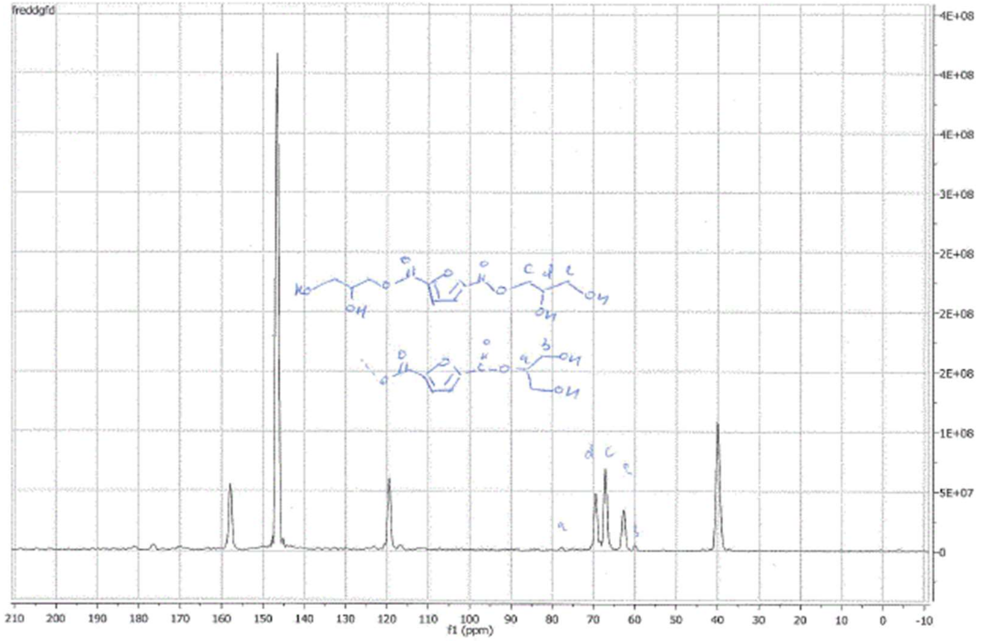
fredddgfd
f1 (ppm)

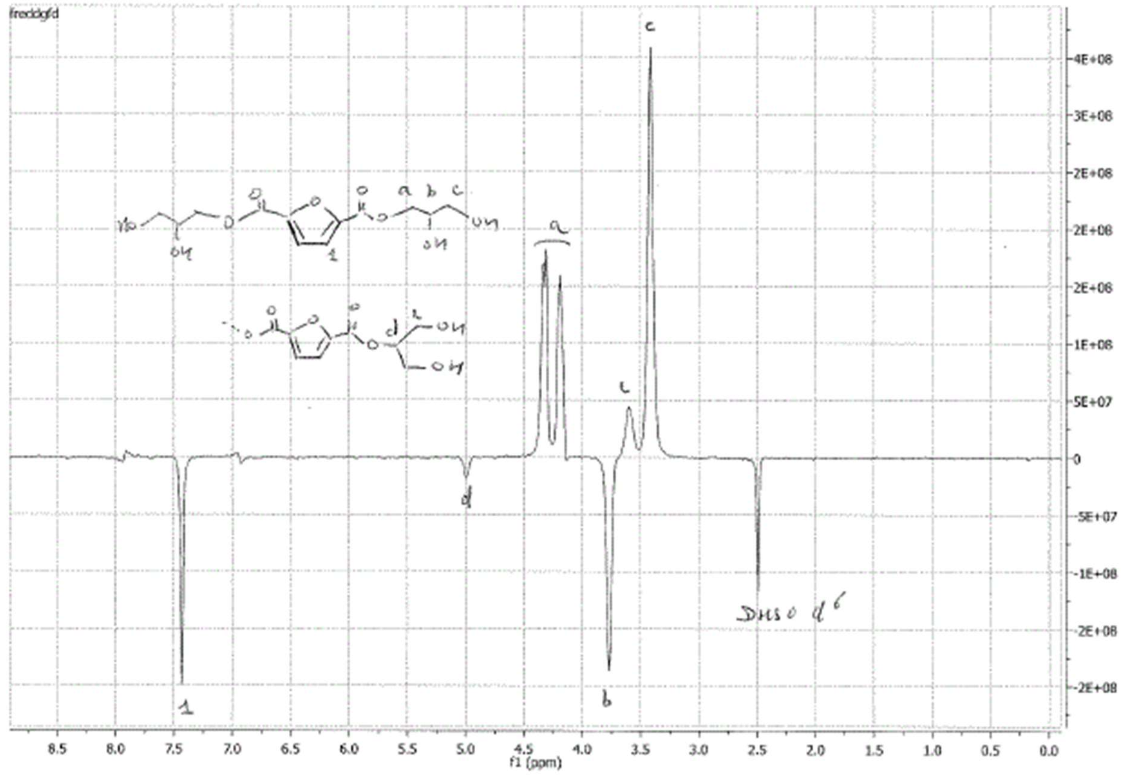
fredddgfd
f1 (ppm)
DMSO d6

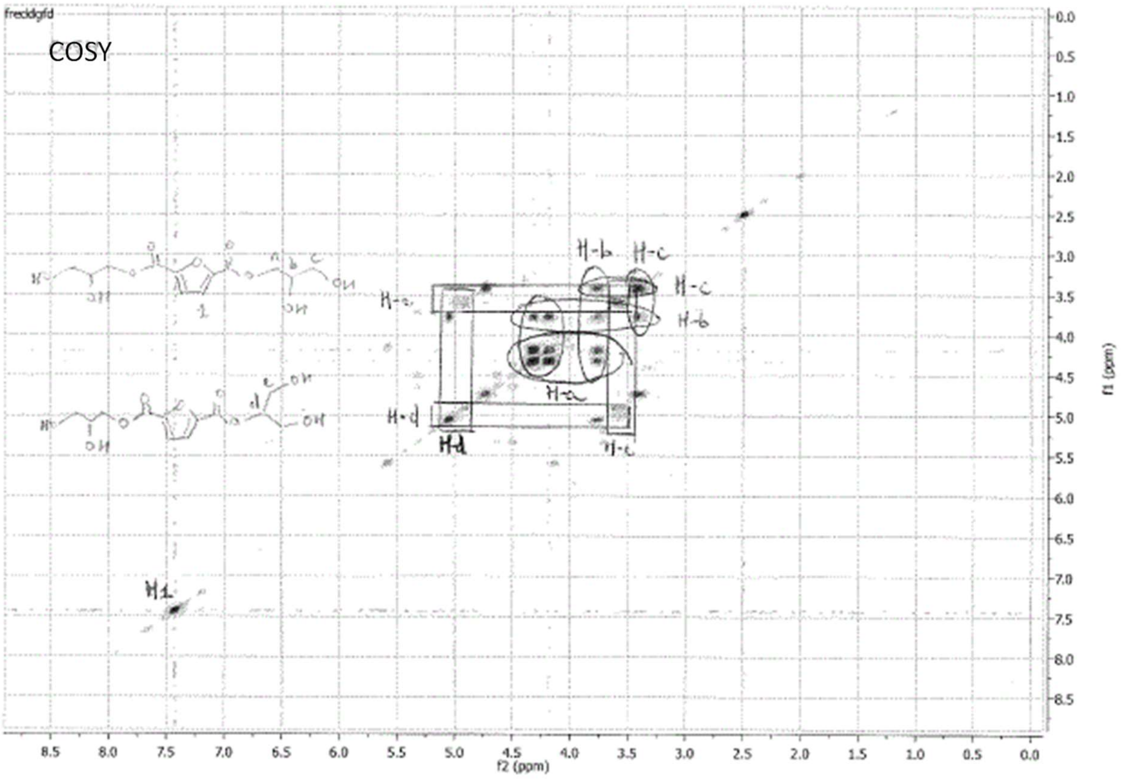

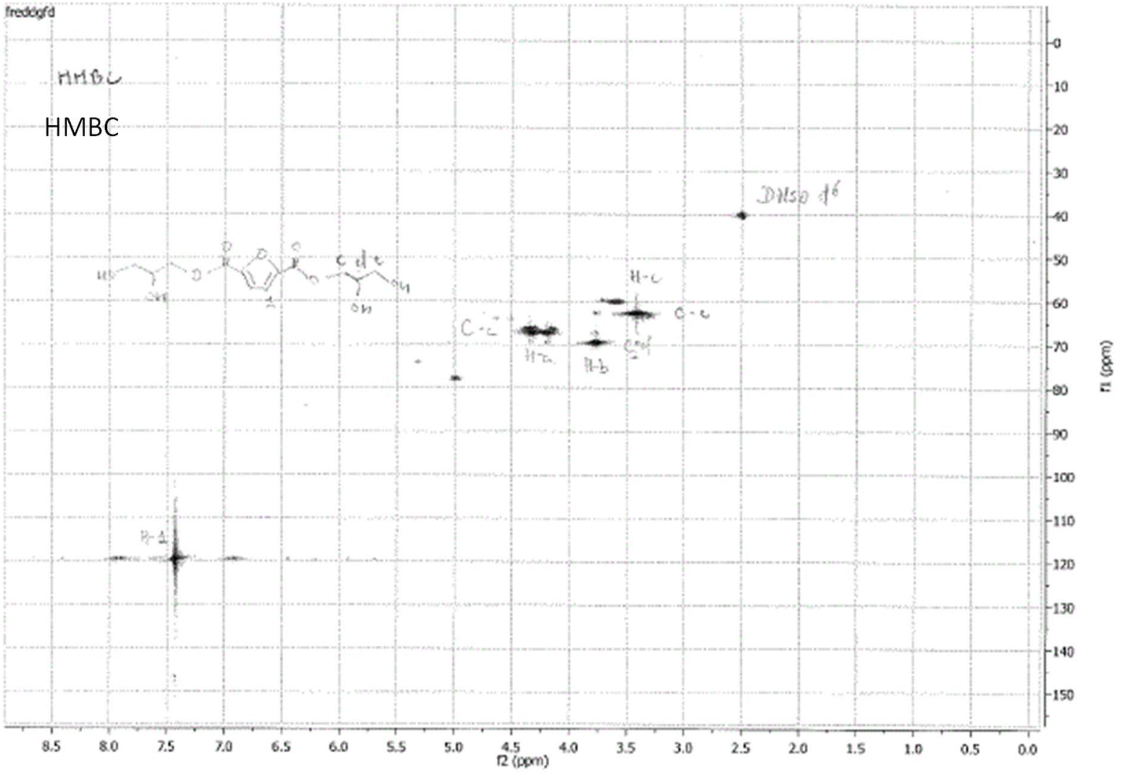

Figure S1. 2D-NMR sequences of BDHPFDC: COSY (^1H-^1H) and HMBC (^1H-^{13}C).

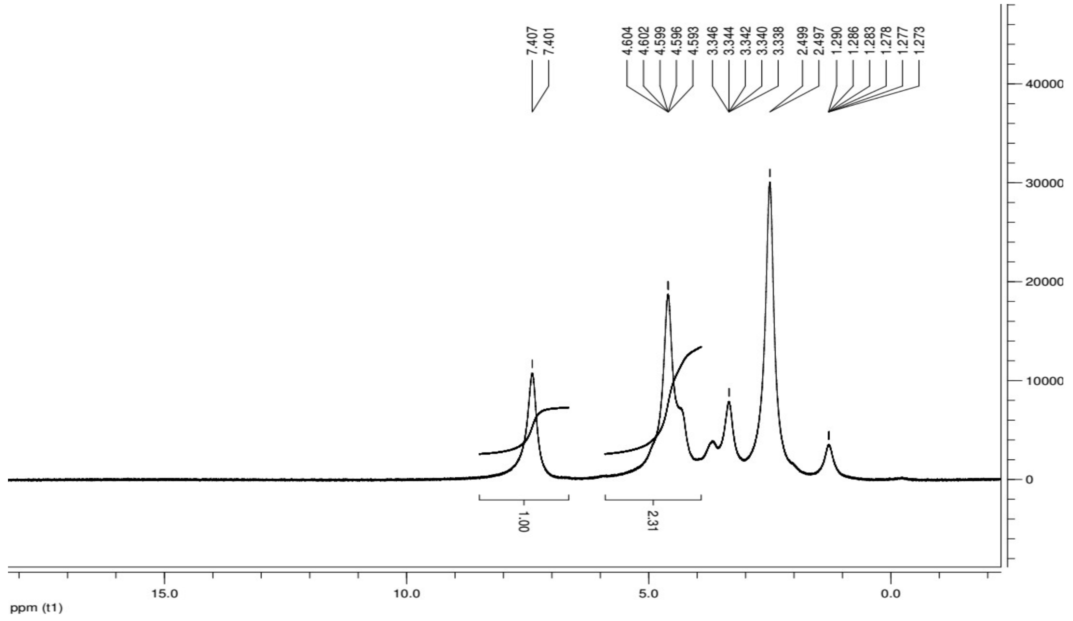

Figure S2. ¹H NMR of PEF in DMSO-d_6 after complete polymerization.

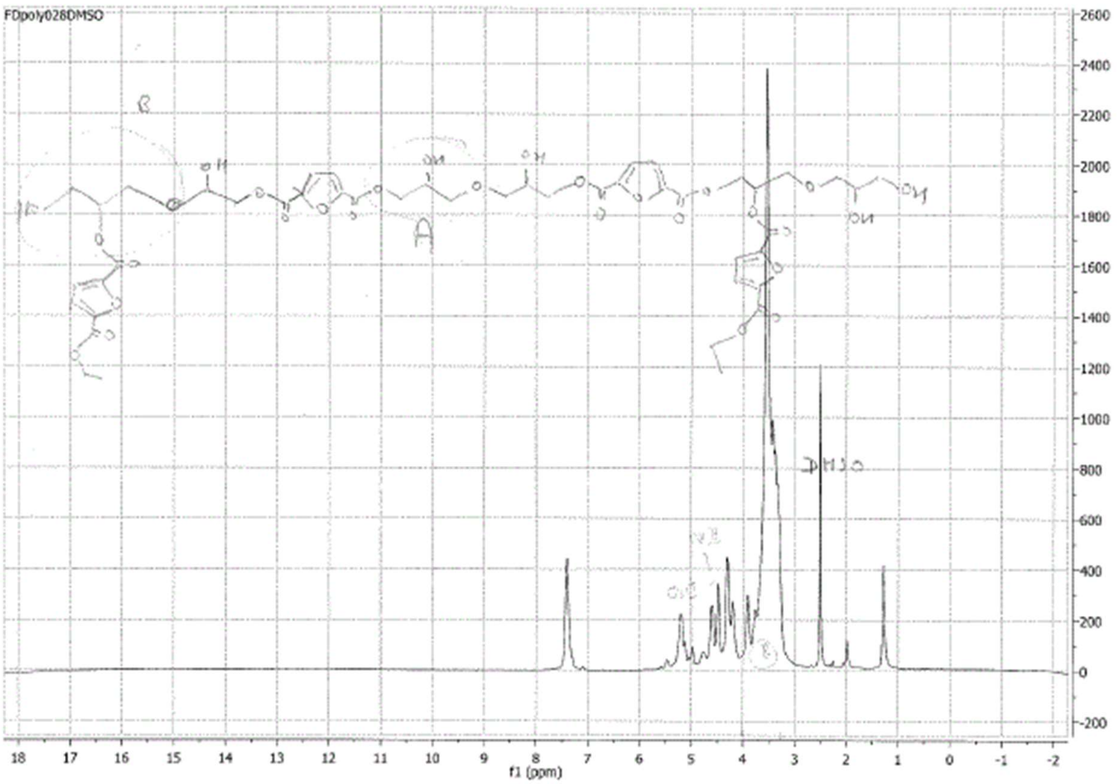

Figure S3. ^{1}H NMR of PDGF after polymerization.

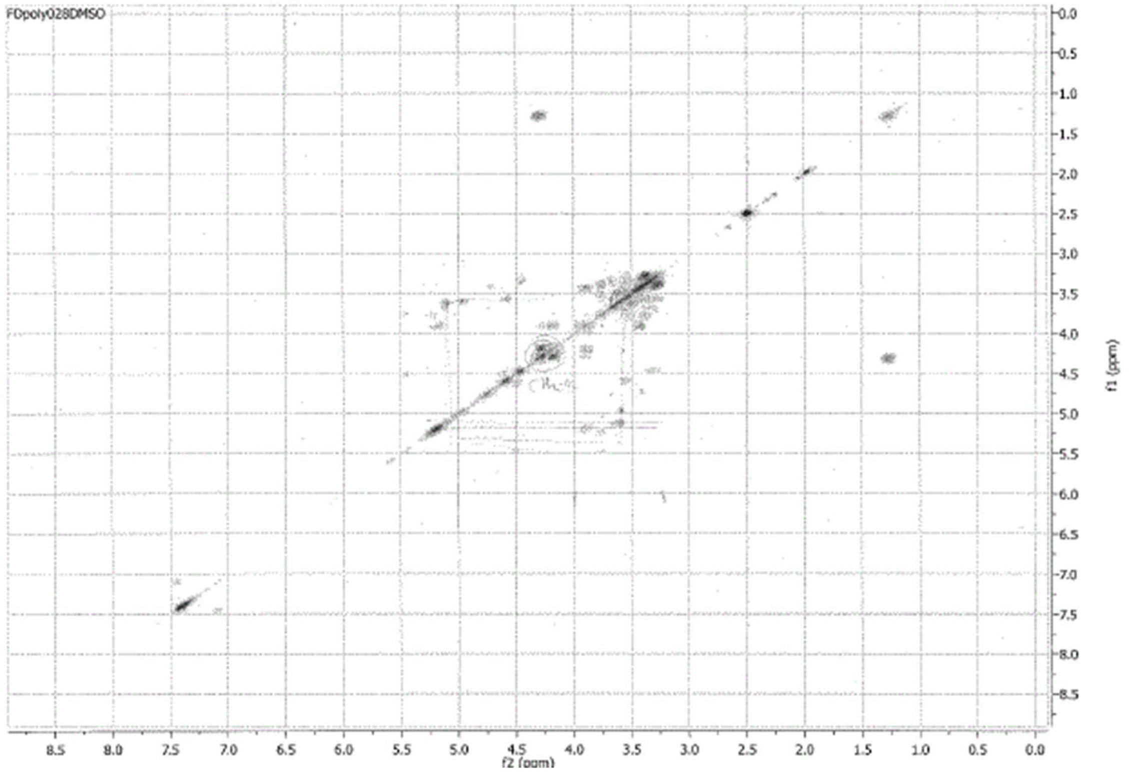

Figure S4. Cosy (1H-1H) spectrum of PDGF.

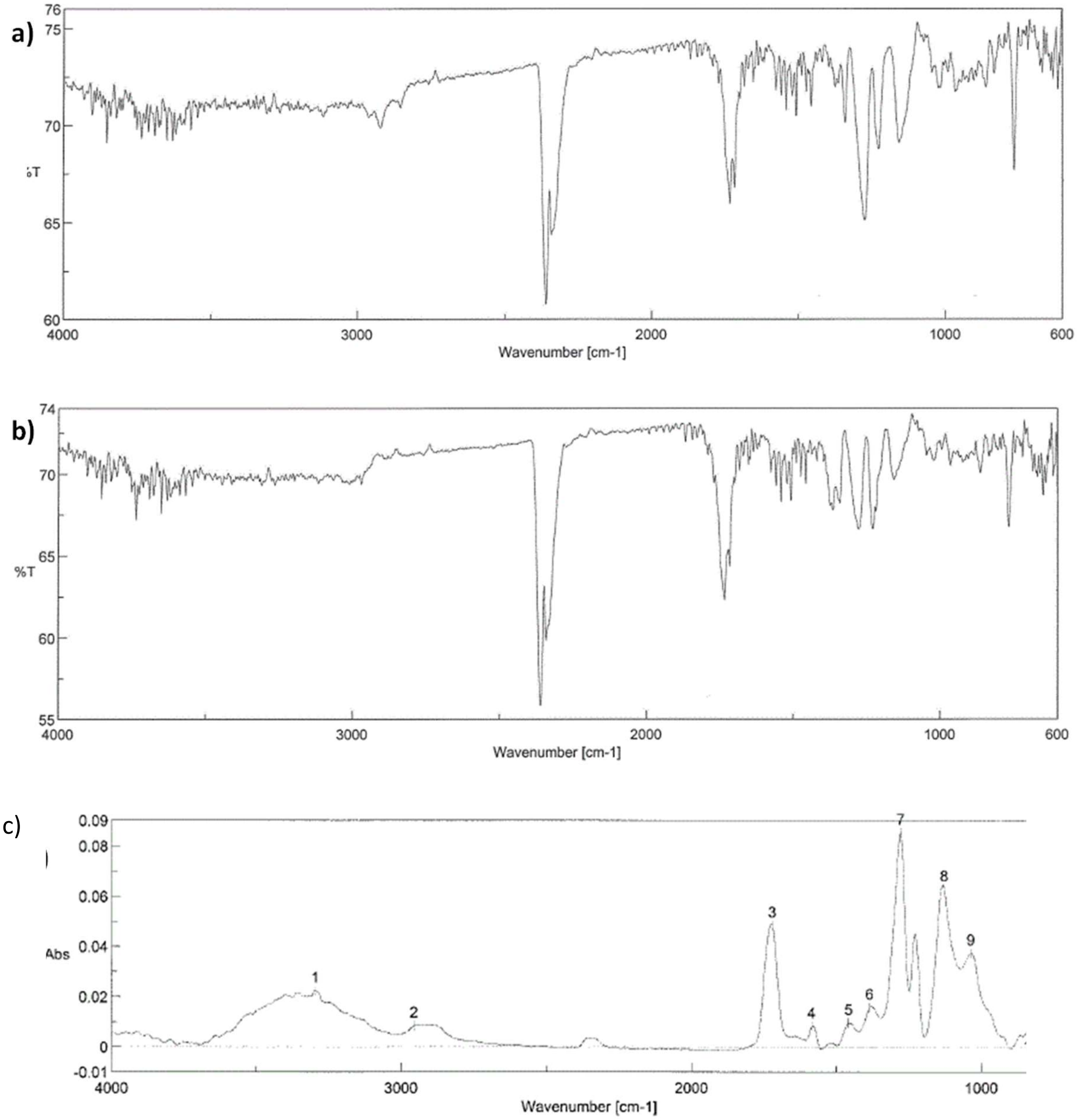

Figure S5. FT-IR spectra of (a) PEF; (b) PHPF and (c) PDGF.

a)

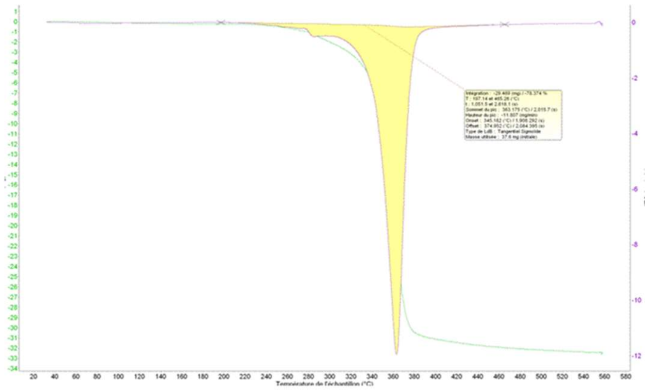

b)

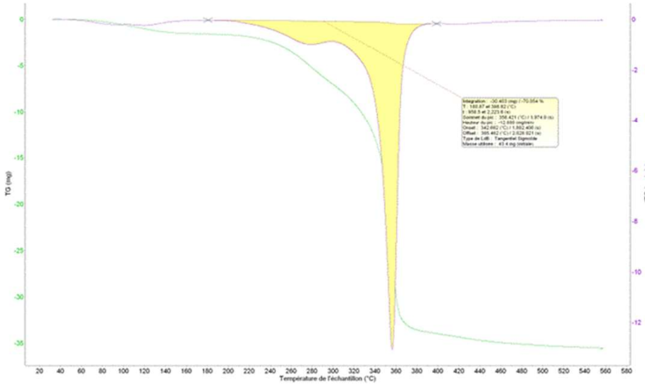

Figure S6. TG-DTG curves of (a) PEF and (b) PHPF.

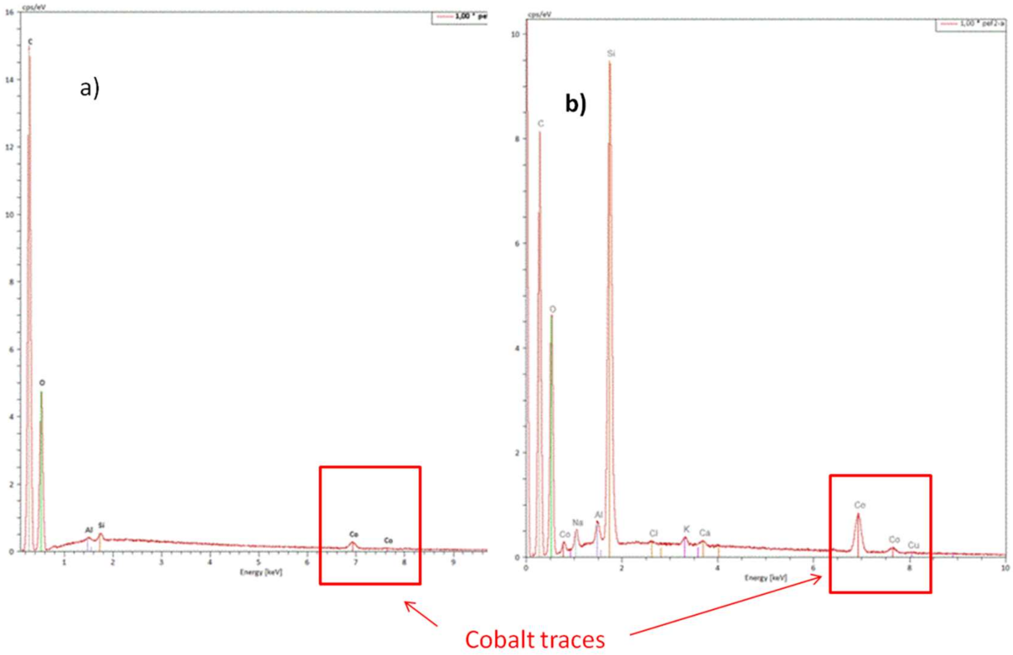

Figure S7. EDX spectra of (a) PEF and (b) PHPF.
